# Fitness, Food, and Biomarkers: Characterizing Body Composition in 19,634 Early Adolescents

**DOI:** 10.3390/nu14071369

**Published:** 2022-03-25

**Authors:** Alina Rodriguez, Katarzyna Korzeniowska, Kamila Szarejko, Hubert Borowski, Michał Brzeziński, Małgorzata Myśliwiec, Leszek Czupryniak, Per-Olof Berggren, Marcin Radziwiłł, Piotr Soszyński

**Affiliations:** 1 Department of Epidemiology and Biostatistics, School of Public Health, Imperial College London, London W2 1PG, UK; 2 Wolfson Institute of Population Health, Queen Mary University of London, London EC1M 6BQ, UK; 3 Department of Pediatrics, Diabetology and Endocrinology, Medical University of Gdansk, 80-210 Gdansk, Poland; kkorzeniowska@gmail.com (K.K.); mysliwiec@gumed.edu.pl (M.M.); 4 PoZdro! Program Scientific Board, Medicover Foundation, 00-807 Warszawa, Poland; k.szarejko@gmail.com (K.S.); hubert.borowski@kosmos.re (H.B.); marcin.radziwill@medicover.pl (M.R.); piotr.soszynski@medicover.pl (P.S.); 5 Department of Pediatrics, Gastroenterology, Allergology & Nutrition, Medical University of Gdansk, 80-210 Gdansk, Poland; brzezinski@gumed.edu.pl; 6 Department of Diabetology and Internal Diseases, Warsaw Medical University, 02-091 Warszawa, Poland; czupryniak@yahoo.co.uk; 7 The Rolf Luft Research Center for Diabetes and Endocrinology, Karolinska Institutet, 171 77 Stockholm, Sweden; per-olof.berggren@ki.se

**Keywords:** adiposity, adolescents, anthropometry, body mass index, fat mass, lifestyle, junk food, obesity, sex differences, PoZdro!, undernutrition, Central and Eastern Europe

## Abstract

Adolescent obesity persists as a major concern, especially in Central and Eastern Europe, yet evidence gaps exist regarding the pivotal early adolescent years. Our objective was to provide a comprehensive picture using a holistic approach of measured anthropometry in early adolescence, including body composition, cardiorespiratory fitness (CRF), and reported lifestyle characteristics. We aimed to elucidate potential sex/gender differences throughout and associations to biomarkers of disease risk for obese adolescents. Methods: Trained nurses measured 19,634 early adolescents (12–14-year-olds), we collected parental reports, and, for obese adolescents, fasting blood samples in four major Polish cities using a cross-sectional developmental design. Results: 24.7% boys and 18.6% girls were overweight/obese, and 2886 had BMI ≥ 90th percentile. With increasing age, there was greater risk of obesity among boys (*p* for trend = 0.001) and a decreasing risk of thinness for girls (*p* for trend = 0.01). Contrary to debate, we found BMI (continuous) was a useful indicator of measured fat mass (FM). There were 38.6% with CRF in the range of poor/very poor and was accounted for primarily by FM in boys, rather than BMI, and systolic blood pressure in girls. Boys, in comparison to girls, engaged more in sports (t = 127.26, *p* < 0.0001) and consumed more fast food (t = 188.57, *p* < 0.0001) and sugar-sweetened beverages (167.46, *p* < 0.0001). Uric acid, a potential marker for prediabetes, was strongly related to BMI in the obese subsample for both boys and girls. Obese girls showed signs of undernutrition. Conclusion: these findings show that overweight/obesity is by far a larger public health problem than thinness in early adolescence and is characterized differentially by sex/gender. Moreover, poor CRF in this age, which may contribute to life course obesity and disease, highlights the need for integrated and personalized intervention strategies taking sex/gender into account.

## 1. Introduction

Health in Central and Eastern Europe (CEE) lags behind that of the West [1,2,3,4], especially for noncommunicable diseases (NCD) [5,6]. Obesity, a major risk factor for NCDs [7] is particularly problematic in Poland [8,9,10,11] though, based on limited data, some improvements have been noted only among boys [11,12]. The United Nation’s Global Strategy targets adolescence [13] as a time to identify vulnerabilities before health risks become entrenched. However, the upward trend in diabetes [7], may leave adolescents, the next generation of soon-to-be adults, facing risk factors and a health gap similar to those of their parents’.

The clinical utility of linking adolescent body mass index (BMI) with adult BMI and disease [14] is hampered because adolescence is defined using very wide age ranges (e.g., 10–24 years) [15]. Early adolescence is marked by rapid growth [16], peak height velocity, and accelerated gains in fat and muscle mass [17]. This period may thus be decisive, as percentage fat mass (FM) and fat-free mass (FFM) show differential associations with NCD risk in adults [18]. Energy balance behaviors need to be considered [19,20]. Because early adolescence is generally less studied and developmentally critical, there is a need to focus on this period, deemed as the second opportunity to set a healthy trajectory for the lifespan [15,21], with a more holistic approach.

This study sets out to provide a systematic and comprehensive picture of anthropometry that covers the spectrum from underweight to obesity among early adolescents aged 12–14 years. Using data from a large-scale screening program for future diabetes prevention, “PoZdro!”, we obtained anthropometric measurements (weight, height, and waist and hip girths), body composition (FM and FFM), and cardiorespiratory fitness (CRF), along with parental reports of lifestyle including consumption of low-nutrient foods for nearly 20,000 pupils. The aim was to characterize the prevalence of adolescent risk factors and examine sex-specific patterns. Specifically, we aimed to clarify the utility of BMI classifications as reflecting FM/FFM and to identify factors associated with CRF in this age group, including lifestyle behaviors. For obese adolescents, we provided a sex-specific analysis of biomarkers of cardiometabolic disease and assessment of the double burden of obesity, i.e., simultaneous undernutrition.

## 2. Materials and Methods

### 2.1. Study Population

Eligibility was set at ages from 12 to <14.5 years, Polish-speaking families, in Gdynia, Warsaw, Lublin, and Wrocław between 2014 and 2017 when all schools were sampled. Recruitment took place in schools during general information assembly to which all pupils in lower secondary grades were invited with their parents. Participation was voluntary and not compensated. Data cover clinical examinations conducted by trained research nurses in the schools and a parental questionnaire using a cross-sectional developmental design. The Medical University of Gdańsk Institutional Review Board approved the study. We followed the Strengthening the Reporting of Observational Studies in Epidemiology (STROBE) reporting guideline (see Supplementary Table S1).

### 2.2. Anthropometric Measures and Body Composition

The Tanita Bioimpedance SC240 scale was used to measure weight, FM, and FFM via foot-to-foot bioelectrical impedance while the adolescent had bare feet and light clothing (underwear or physical education uniform). This scale has shown acceptable correspondence to estimated percent body fat in young adolescents as compared to dual-energy X-ray absorptiometry [22]. It is a validated three-compartmental device. The measure of fat-free mass that we report includes water and excludes bone mineral mass. Height was measured with a Mechanical Child and Adult Height Monitor Seca accurate to 1 mm. BMI (kg/m^2^) was used as a continuous variable or classified according to the sex and age-specific cut-offs proposed by the International Obesity Task Force (IOTF) for females/males using six-month age bands [23]. Using a standardized tape measure, waist and hip girths (mm) were taken; the mean of two valid measurements was used. We calculated waist–hip ratio (WHR) and waist-to-height ratio (WHtR) in cm, as measures of central adiposity.

### 2.3. Cardiorespiratory Measures

The average of three blood pressure readings using an Omron M3 blood pressure monitor and an Omron cuff (32–42 cm) while at rest at one-minute intervals was used.

CRF, an index of metabolic and cardiovascular profile, was assessed using the 3 min Step Test [24] which has been found to strongly correlate with VO_2_max and have excellent discriminative validity in a pediatric population [25]. Participants step up and down on a 30.5 cm step at an established rhythm set of 24 steps per minute using a digital metronome KORG Micrometro. Heart rates (HR) are monitored prior to the test, during the 3 min of exercise load (step-test), immediately after, and one minute post [24] using the electronic analyzer, TM 100 Pro Tech Med Heart Rate Monitor. Post-effort HR values were analyzed and recorded for one minute. Scores were automatically generated based on the arithmetic mean of HR following Jacks et al. formula [26] to categorize CRF as very poor, poor, satisfactory, good, very good, or excellent.

### 2.4. Blood Sampling for Adolescents with BMI ≥ 90th Percentile

Overnight fasting blood samples were collected in the morning and stored at –70 °C until analyzed for key metabolic biomarkers all used as continuous variables: fasting serum glucose, triglycerides (TG), high-density (HDL) and low-density lipoprotein cholesterol (LDL), total cholesterol, and uric acid (UA) for obese adolescents. To assess the double burden of obesity and undernutrition, we examined hemoglobin and mean corpuscular hemoglobin concentration (MCHC) as indices of iron. The MCHC measures the amount of hemoglobin adjusted for the effect of cell size. We used MCHC as an index of iron availability in relation to potential anemia or iron deficiency [27]. Iron deficiency is associated with long-term inadequate iron intake and poor iron absorption or utilization, and may result in anemia [28].

### 2.5. Parental Questionnaire

All parents reported adolescent lifestyle behaviors using a questionnaire designed for this study to give a rapid overview of health and health-related behaviors. An index of high junk food consumption was computed as consumption ≥3 times per week of junk food snacks, fast food, and sugar-sweetened beverages. Parents reported the number of hours spent on active sport per week in addition to school physical education and hours per day using a screen device, e.g., computer/TV.

The family obesogenic environment was assessed by parental report of height and weight converted into BMI. Parents reported whether they believed family members were overweight/obese as yes/no. Parents indicated whether they perceived the adolescent as severely underweight, underweight, normal, overweight, or obese. Parents rated their perception of the adolescent’s health (very good, good, satisfactory, poor, “I don’t know”). To assess the extent that parents had insight into their adolescent’s health behaviors, we created a dummy variable where all “I don’t know” or missing responses were coded as 0 and an answer coded as 1 and summed (range 0–10).

Maternal and paternal education were dichotomized as primary, vocational, secondary (coded as 1) and any post-secondary or higher education as 2. We used parental education as covariates.

### 2.6. Statistical Analyses

All analyses were stratified by sex, as our aim was to study sexual dimorphism which is fundamental in early adolescence. We used R version 4.1.0 with an a priori level of significance set at *p* < 0.05 (two-sided) and 95% confidence intervals (CI). We examined differences between groups by *t*-test or Wald χ^2^ and χ^2^ goodness of fit using equal proportions as the expected values. We used Cochran Armitage Test to test trends across 6-month age groups. Multiple regression analyses were used to examine associations while adjusting for age, city, and maternal education (paternal education was excluded due to missingness). Alternate models were tested using either BMI vs. IOTF BMI and FM vs. FFM. Potential multicollinearity was assessed via pairwise Pearson, Spearman, or Point Biserial correlation coefficients. Only variables with the strongest associations were selected for modelling FM/FFM. Backward selection was used in multiple regression analysis to identify the impact of variables in the prediction of CRF. Correlations and multiple regression were used to evaluate family and lifestyle factors. Missing values were pairwise deleted. Biomarkers with skewed distributions were log transformed prior to analyses.

## 3. Results

### 3.1. Sample Description

We analyzed valid data from 19,634 adolescents (Table 1) of whom 2886 (14.7%) had BMI ≥ 90th percentile. The population of adolescents in the first years of secondary school was 31,939, however due to changes in school entrance ages during data collection, not all were eligible. Proportions of males to females did not differ within cities (Table 1). Most parents’ education was beyond secondary school (mothers 57.4%; fathers 44.1%), paternal education did not differ by city (χ^2^ = 7.322, *p* = 0.062) however, maternal education was highest in the capital Warsaw (χ^2^ = 11.68, *p* = 0.008). The distribution of IOTF BMI differed by city (χ^2^ = 32.037, *p* = 0.006) such that the prevalence of overweight and obesity was the lowest in Wrocław and the highest in Lublin. Parents’ perception of adolescent health did not differ by city (χ^2^ = 3.937, df = 3, *p* = 0.27).

### 3.2. Sex Differences in Adiposity

**Figure 1:**
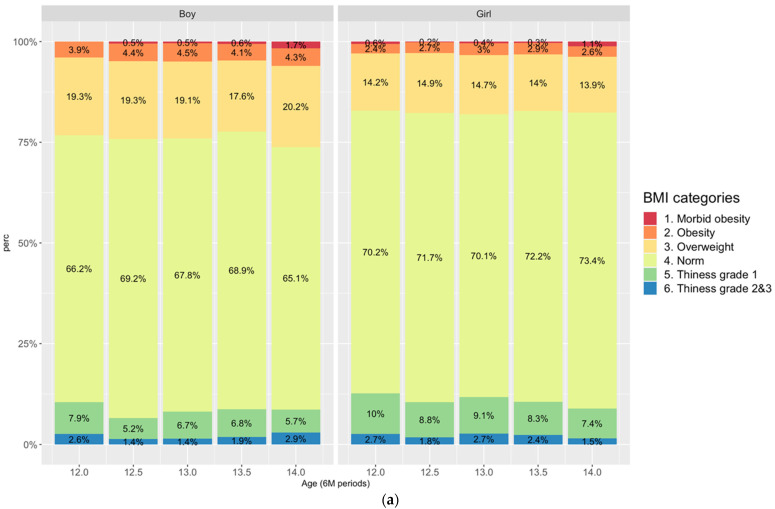

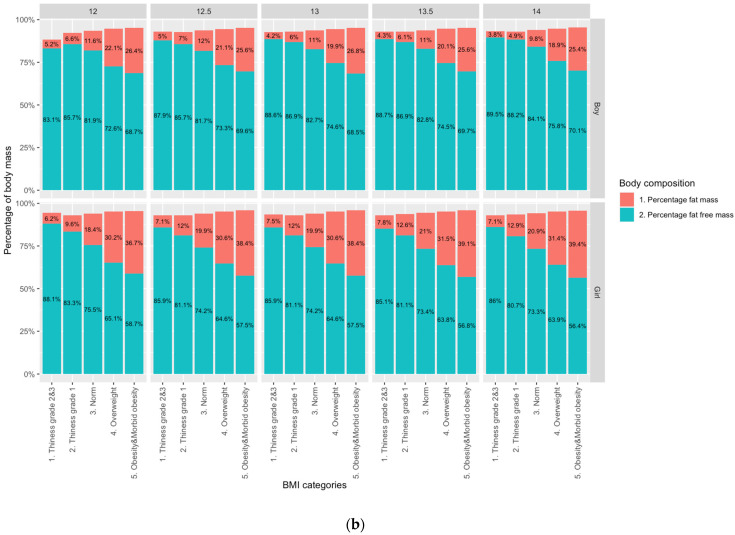
(**a**) IOTF BMI categories by sex and age; (**b**) Body composition by IOTF BMI categories, sex, and age.

We present a graphic analysis of IOTF BMI distributions by age and sex in Figure 1a. This analysis was intended to identify sex and age differences. Both boys and girls showed a pattern of increasing prevalence of high BMI with age. There were no boys in the morbid obese category at age 12 as compared to 1.7% at age 14 when 26.2% were overweight/obese. Trend test showed a significant increase in morbid obesity among boys with age (*p* = 0.001). For girls, the trend test was nonsignificant; a total of 17.2% were overweight, obese, or morbidly obese (0.6%) at age 12, compared to 17.6% and 1.1%, respectively, by age 14. There was a similar albeit downward and nonsignificant trend in prevalence of thinness in boys, but for girls, the trend test showed a significant decrease in prevalence of thinness with increasing age (*p* = 0.01). For both sexes and across ages, the prevalence of overweight and obesity was nearly or more than double that of thinness.

Figure 1b shows the distribution of body composition as total FM/FFM (exclusive of bone mineral mass) across ages for each sex within the IOTF BMI categories. For boys, FM at every IOTF BMI category was lower than that for girls. FM across the IOTF BMI categories in girls was relatively stable across age with the thinnest girls having 6.2–7.1% FM at ages 12 and 14, respectively, which contrasted with 36.7 and 39.4% among 12 and 14-year-old girls in the obese category.

To identify anthropometric measures that adequately capture adiposity, we first examined the magnitude of the associations between FM and BMI (r = 0.77, CI: 0.76–−0.77) vs. IOTF BMI (r = 0.61, CI: 0.60–−0.61) and WHtR (r = −0.69, CI: −0.70–−0.68) vs. (WHR: r = 0.29, CI: 0.28–−0.31). We tested relative predictive value of BMI versus IOTF BMI on FM alternatively FFM and included WHtR, adjusting for age, maternal education, and city. Table 2 shows that relative to IOTF BMI the models using BMI were superior in predicting both FM and FFM as indicated by percent of variance explained and root mean squared error (RSME) for both boys and girls.

### 3.3. CRF

Our aim was to understand the contemporaneous variables related to CRF. By using backward selection multiple regression analysis, we examined the best predictors of CRF from anthropometric measures (BMI, WHtR, FM, or FFM), lifestyle factors (sports, sedentary behavior, and junk food), and adjusted for age, maternal education, and city. Table 3 shows that models using either FM or FFM were relatively equal according to RSME. CRF for boys was best predicted by FM/FFM, followed by sports activity, with BMI having a lesser impact for both sexes. Systolic blood pressure was the best predictor for girls. Despite girls having greater FM than boys, they were more likely to have better CRF scores (Table 1).

### 3.4. Lifestyle and Family

Our objective was to assess sex differences in obesogenic lifestyle and family environment.

Table 1 shows that boys had higher consumption of fast food, sugar-sweetened beverages, and sedentary time. Nonetheless, boys spent more time in active sports than girls.

We examined the potential transgenerational impact of obesity and found a positive association between the number of relatives who were overweight/obese (36.4% reported at least one relative) and adolescent BMI (rho = 0.24, CI: 0.23–−0.26). The sex-specific cross-correlations between adolescent and parental BMI did not indicate sex-specific parent-of-origin associations (r’s = 0.22–0.30; overlapping CIs). Most parents’ (85.9%) perceptions were accurate, i.e., matched measured adolescent IOTF BMI. However, 1140 parents (6.48%) underestimated true adolescent overweight and obesity, while 1340 (7.62%) wrongly believed their underweight adolescent had normal weight (daughters: 8.67%; sons: 6.51%). We explored whether parental BMI influenced their perceptions and found that both maternal and paternal overweight/obesity was associated with an underestimate of adolescent BMI (χ^2^ = 6.34, *p* < 0.05). We hypothesized that lack of parental insight regarding adolescent lifestyle behaviors would be associated with adolescent obesity. Results of MANOVA (entering adolescent, maternal, and paternal BMI) showed that significant lack of insight was best predicted by adolescent BMI for both sexes, and additionally for girls by paternal obesity. In other words, parents were more likely to lack insight when adolescents were overweight/obese.

### 3.5. Biomarkers of Disease Risk

We were interested in identifying the degree to which anthropometric measures (BMI, FM, and WHtR) were associated with cardiometabolic and iron biomarkers among the subsample of adolescents at ≥90th percentile BMI stratified by sex and adjusting for age, maternal education, and city. Table 4 shows a sex-specific pattern with boys showing more signs of disease risk than girls. There was little or no evidence of FM or central obesity being associated with a biomarker. Instead, BMI was largely associated with the biomarkers, especially UA. MCHC was negatively associated with higher BMI only in girls, indicating potential double burden of obesity and undernutrition.

## 4. Discussion

We examined anthropometry, body composition, CRF, and family factors inclusive of junk food consumption for nearly 20,000 12–14-year-olds and additionally biomarkers of disease risk in the sub-sample of nearly 3000 obese early adolescents. Previous research has clumped wide age ranges together [12,29,30]. leaving a gap in understanding for the period of early adolescence, which is pivotal from both biological and psychosocial perspectives. Our findings showed clear sex differences across the spectrum of underweight to obesity and suggested that overall boys were less healthy than girls.

Our results showed that the prevalence of overweight/obesity was two-fold higher than that of underweight, already surpassing the 2030 predictions for child and adolescent obesity [31]. The use of BMI in studies of children and adolescents is disputed because of concerns that it may not accurately reflect adiposity—the true risk factor for disease [32,33,34]—nor differentiate between FM and FFM [32,35,36,37,38]. We directly compared models of BMI and IOTF BMI in relation to FM and FFM and found that they were comparable. BMI (on a continuous scale) explained a slightly greater portion of variance in both FM and FFM. Together with WHtR, the models explained as much as 89% of the variance in FM and FFM, thus highlighting the utility of BMI and WHtR for both clinical screening and in epidemiological studies.

CRF is supposedly a direct consequence of BMI and physical activity [39]. Instead, we found that FM or FFM made marked impacts on CRF, especially for boys. The order of factors differed in a sex-specific manner. Physical activity and FM/FFM were important for CRF in boys, presumably because they were more physically active, while girls, congruent with previous studies [40,41], were more sedentary. These results suggest that increasing sports activity should be targeted to improve CRF with additional benefit for obesity, especially for girls to counteract the social tendency towards sedentary behavior, though junk food consumption did not associate with CRF in either sex. Most recommendations include 60 minutes of daily moderate-to-vigorous physical activity for adolescents [42], but we found that approximately 1/3 and 1/5 of boys and girls, respectively, met this goal. We found using a valid measure of CRF that an alarmingly high prevalence (nearly 40%) of adolescents scored poor or very poor, which suggests that a large portion of adolescents will probably not engage in sports due to feeling exhausted. Consequently, poor CRF at this stage in life can set a trajectory of sedentary behavior and obesity.

Shared genetic determinants between offspring and parental BMI are to be expected [43], as are family factors. Our intention was to explore parents’ perceptions and own BMI. We added to previous findings that parents are likely to underestimate obesity [44] by showing that parents’ own high BMI contributed to underestimating adolescents’ true weight, suggesting that overweight/obesity may go unrecognized particularly in these families. Conversely, normal-weight girls were more likely than boys to be perceived as overweight, suggesting that parents hold an ideal for female beauty as slim and may predispose girls to depressive symptoms [45]. Indeed, parents have been found to tease their youngsters for overweight [46]. Lack of parental insight, which is related to strained family functioning and adolescent risk-taking behavior [47,48], was associated with adolescent overweight/obesity. These finding underlines the need to include families in interventions.

We investigated biomarkers of risk for cardiometabolic disease and iron deficiency in obese adolescents. We found that central adiposity and FM were not related in a meaningful way to the biomarkers. Contrariwise, BMI explained a small yet significant amount of variance in most biomarkers that have been previously reported for adults [49]. UA, the end-product of purine metabolism, was recently confirmed in a sample of 5–17-year-olds (without differentiating ages) as a risk factor for prediabetes [50]. We extended those findings by showing sex-specific associations among early adolescents that point to girls, who we found carry more fat mass, as being more vulnerable to prediabetes, though in neither sex was fasting glucose associated. Obese girls, according to MCHC, had low iron, which has ramifications not only for vitality, but also for growth and development. It is worth noting that substantial variance was accounted for by city of residence, which suggests socioenvironmental inequalities play a role in obesity [51].

The cities were selected to represent a cross-section of urban centers covering the four cardinal points: Gydnia on the north coast (population = 244,969), Lublin, southeast (population = 338,586), Wrocław, southwest (population = 641,928), and Warsaw, the capital in the center of Poland (population = 1.8 million). The cities are diverse with respect to within-city income inequalities as reflected by large Gini coefficients (Lublin = 0.52; Gydnia = 0.54, Wrocław = 0.54, Warsaw = 0.57) [52], and according to the index of deprivation, the greatest risk of deprivation is in Lublin, which ranks in the highest tier, while the other three cities are in the lowest [52]. Nonetheless, we found no differences regarding paternal education or parental perceptions of adolescent health. Though all analyses were adjusted for city of residence, this does not fully adjust for residual confounding and merits further study. There are many reasons behind the health of a city, including availability of health services, pollution, availability of nutritious and junk food, walkability, safety, deprivation, etc. These are topics worth delving into in studies specifically designed to look at these questions and are beyond the scope of the present investigation.

Intense sociopolitical changes in Poland have been reflected at par by fluctuations in child and adolescent BMI [53,54] and accelerated pubertal timing [10]. These secular trends in higher standard of living, also observed in CEE [55], are likely to be underpinned by societal norms towards sedentary lifestyle and caloric-rich or ready-made processed foods [3,9]. Our results confirm a preference for junk food by boys. Our results provide inferences for emerging economies that are likely to experience problems with obesity, especially in children/adolescents [56,57], and mounting healthcare costs that follow.

Previous studies provide conflicting evidence on whether prevalence of obesity differs by sex in children and adolescents [58,59,60,61,62]. We reported higher prevalence of overweight/obesity in boys as compared to that in girls, though girls, at every age group, carried between 10–14% more fat mass than boys of the same BMI. FM accretion in girls is not only driven by hormonal differences, but also by lifestyle, which can result in gender-specific associations with energy metabolism. The early adolescent growth spurt is one of the most rapid periods of development, and so perturbations may have lasting health and developmental consequences [63,64,65]. Adolescent obesity is related to neurodevelopmental problems [66,67] which can lead to risk-taking behavior and may have an impact on intergenerational obesity. Polish mothers are among the youngest in Europe [68], thus it is conceivable that obese adolescent girls will enter pregnancy overweight/obese and pose risk to their offspring for neurobehavioral problems and obesity [69,70,71].

This study has several limitations. We did not have data available to calculate the exact response rate. However, parental age and education suggest that families were socioeconomically representative of the recruitment area. We recruited in cities, and further work is needed to assess adolescents living in rural areas or in smaller towns. Recruitment took place during general school assemblies in all the schools serving pupils in early adolescence. Not all adolescents in the schools were eligible, ages of 12–14 years, which we targeted to ensure that the participants had reached pubarche. Precise information on pubertal development would have been useful for analyzing metabolic changes [72]. However, our age range falls within the overall genetic architecture regulating pubertal initiation [73]. Further, the parental questionnaire was designed to suit the population under study and was specifically intended to give an overview without burdening participants with long, detailed questions, e.g., a food frequency questionnaire, which would have provided more detailed knowledge. This decision was taken to increase participation as this was meant to be a large-scale study and to give clues on what aspects need further and more detailed data in the future. Despite its uncomplicated format, some parents reported lack of knowledge or insight regarding their adolescents’ lifestyle behaviors regarding food consumption. This finding supports future efforts in enlisting adolescents to answer such questions themselves, nonetheless, parental lack of insight was informative as it was associated with adolescent overweight and obesity.

These limitations are offset by the large size of our study that provided the opportunity to examine sex-specific associations within a narrow age band during the crucial early adolescent period that can set enduring health [74,75] and educational [76] and economic trajectories [77]. It has been encouraged that a more holistic approach be taken to increase our understanding of obesity [78] and adolescence [15,79,80]. Therefore, we described contemporaneous factors to fill the knowledge gap for anthropometric measures, CRF, and family factors in relation to adolescent weight.

## 5. Conclusions

We report distinct sex/gender differences in clinically measured BMI, FM/FFM, CRF, and reported lifestyle family factors and how these factors are inter-related during early adolescence. Knowledge of these differences can aid clinical practice and inform policymakers.

## Figures and Tables

**Table 1:** Characteristics of the PoZdro Study Population ^a^ with sex-specific analyses ^b^.

Characteristic	All Participants *N* = 19,634	Males *N* = 9556 (48.7%)	Females *N* = 10,078 (51.3%)	Male-Female Comparison χ^2^ or *t*-Test	*p*-Value
Study Site (*n*, %)				10.37	*p* = 0.0157
Gdynia	3914 (19.9)	1898 (48.5)	2016 (51.5)	3.56	*p* = 0.0593
Lublin	4709 (24.0)	2339 (49.7)	2370 (50.3)	0.20	*p* = 0.6514
Warsaw	6662 (33.9)	3290 (49.4)	3372 (50.6)	1.01	*p* = 0.3151
Wrocław	4349 (22.2)	2029 (46.7)	2320 (53.3)	19.47	*p* < 0.0001
Age (*n*, %)				27.936	*p* < 0.0001
12.0	606 (3.1)	248 (40.9)	358 (59.1)	19.97	*p* < 0.0000
12.5	2271 (11.6)	1047 (46.1)	1224 (53.9)	13.80	*p* = 0.0002
13.0	7598 (38.7)	3677 (48.4)	3921 (51.6)	7.84	*p* = 0.0051
13.5	7411 (37.7)	3701 (49.9)	3710 (50.1)	0.01	*p* = 0.9167
14.0	1748 (8.9)	883 (50.5)	865 (49.5)	0.19	*p* = 0.6668
Height, cm	163.5 ± 7.8	165.4 ± 8.7	161.8 ± 6.4	33.02	*p* < 0.0001
Weight, kg	54.1 ± 12.1	55.6 ± 13.1	52.6 ± 10.7	17.46	*p* < 0.0001
BMI ^c^	20.1 ± 3.6	20.2 ± 3.8	20.0 ± 3.5	2.75	*p* = 0.0060
IOTF ^d^ BMI, %				139.84	*p* < 0.0001
Thinness grade 2 & 3	2.0	1.7	2.4	15.01	*p* < 0.0001
Thinness grade 1	7.6	6.5	8.5	38.72	*p* < 0.0001
Normal	68.9	67.1	70.6	38.24	*p* < 0.0001
Overweight	17.4	19.6	15.4	29.58	*p* < 0.0001
Obesity and Morbid obesity	4.1	5.1	3.2	33.79	*p* < 0.0001
Waist girth, cm	69.06 ± 9.17	71.09 ± 9.70	67.12 ± 8.19	30.80	*p* < 0.0001
Hip girth, cm	86.94 ± 8.77	86.36 ± 9.16	87.48 ± 8.36	8.93	*p* < 0.0001
Waist–hip ratio (WHR)	0.795 ± 0.068	0.823 ± 0.64	0.768 ± 0.061	61.92	*p* < 0.0001
Waist-to-height ratio (WHtR)	0.422 ± 0.052	0.430 ± 0.054	0.415 ± 0.048	20.18	*p* < 0.0001
Percent Fat Mass (FM)	17.6 ± 8.3	13.3 ± 6.8	21.7 ± 7.5	81.89	*p* < 0.0001
Percent Fat-Free Mass (FFM)	76.5 ± 7.8	80.6 ± 6.5	72.7 ± 6.9	83.15	*p* < 0.0001
Systolic blood pressure	115.1 ± 11.4	116.9 ± 11.8	113.5 ± 10.7	20.61	*p* < 0.0001
Diastolic blood pressure	69.3 ± 8.0	68.6 ± 8.0	70.0 ± 7.8	12.63	*p* < 0.0001
Cardiorespiratory Fitness (CRF) ^e^ Classification ^f^ %				163.7	*p* < 0.0001
Excellent	2.8	2.6	3.0	1.71	*p* = 0.1913
Very good	9.7	9.9	9.5	0.39	*p* = 0.5322
Good	19.3	17.7	20.9	19.94	*p* < 0.0001
Satisfactory	29.6	28.1	31.2	13.32	*p* = 0.0002
Weak	31.8	32.5	31.0	2.46	*p* = 0.1169
Very weak	6.8	9.2	4.4	125.9	*p* < 0.0001
Heart rate (HR) post exertion	126.2 ± 15.9	121.3 ± 15.2	131.2 ± 15.0	40.21	*p* < 0.0001
Perceived adolescent BMI (parental report), %				175.61	*p* < 0.0001
severe underweight	1.8	2.2	1.4	10.71	*p* = 0.0011
underweight	13.1	15.4	11.0	42.10	*p* < 0.0001
normal	67.2	62.4	71.7	117.80	*p* < 0.0001
overweight	14.3	15.9	12.7	16.57	*p* < 0.0001
obesity	3.6	4.0	3.2	5.53	*p* = 0.0210
Reported maternal BMI	23.90 ± 4.03	23.87 ± 4.05	23.92 ± 4.01	0.80	*p* = 0.4263
Reported paternal BMI	26.91 ± 3.79	26.91 ± 3.76	26.90 ± 3.83	0.26	*p* = 0.7942
Fast-food consumption, %				188.57	*p* < 0.0001
>5 times per week	0.3	0.4	0.3	2.48	*p* = 0.1151
3–5 times per week	1.3	1.6	0.9	14.07	*p* = 0.0002
1–3 times per week	49.8	54.5	45.3	33.18	*p* < 0.0001
no consumption	42.9	37.8	47.8	151.53	*p* < 0.0001
unknown	5.7	5.6	5.7	1.27	*p* = 0.2594
Junk food snacks, %				9.74	*p* = 0.08
few times per day	13.2	12.5	13.9	15.85	*p* = 0.0001
≤1 per day	27.5	27.7	27.2	2.07	*p* = 0.1504
few times per week	46.1	46.2	45.9	5.92	*p* = 0.0150
≤1 per week	11.3	11.7	11.0	0.03	*p* = 0.8579
no consumption	0.7	0.7	0.7	0.03	*p* = 0.8608
unknown	1.1	1.1	1.2	1.27	*p* = 0.2603
Sugar-sweetened beverages, %				167.46	*p* < 0.0001
≥5 times per week	15.5	17.0	14.1	11.11	*p* = 0.0009
3–5 times per week	16.1	17.2	15.0	3.44	*p* = 0.0635
1–3 times per week	41.2	42.8	39.8	0.25	*p* = 0.6195
no consumption	25.8	21.4	29.8	167.43	*p* < 0.0001
unknown	1.4	1.6	1.3	1.44	*p* = 0.2304
Number of hours of active sport activity per week, %				319.64	*p* < 0.0001
≥5 h	29.0	34.7	23.7	127.26	*p* < 0.0001
3–5 h	24.3	24.2	24.4	5.17	*p* = 0.02
1–3 h	32.8	30.3	35.2	62.87	*p* < 0.0001
<1 h	13.9	10.9	16.7	139.97	*p* < 0.0001
Sedentary behavior, %				110.62	*p* < 0.0001
>3 h	32.9	36.0	29.9	22.03	*p* < 0.0001
1–3 h	53.6	52.7	54.6	22.01	*p* < 0.0001
<1 h	13.5	11.3	15.5	83.32	*p* < 0.0001

^a^ Frequency and percentages or means and standard deviations; ^b^ χ^2^ Goodness of Fit assuming equal distribution by sex or independent sample *t*-test; ^c^ Body Mass Index, weight/height (meters)^2^; ^d^ International Obesity Task Force sex and age-specific classification for BMI in children and adolescents; ^e^ Step Test; ^f^ CRF classification of VO_2max_ based on Jacks et al., 2012 [25] formula −2.045 + (height in inch × 0.062) + 100 × [1/3 × (HR 1 min + HR 2 min + HR 3 min)/HR baseline] × (−0.411) + (HR baseline × 0.011).

**Table 2:** Association between anthropometric measures and percent fat mass (FM) and percent fat-free mass (FFM).

	FM					FFM				
Models:	Unadj Model Adj *R*^2^	Adjusted ^a^ Model Adj *R*^2^	β	CI	RSME	Unadj Model Adj *R*^2^	Adjusted ^a^ Model Adj *R*^2^	β	CI	RSME
**Testing BMI**										
**Boys**	0.76	0.76			0.032	0.69	0.69			0.035
BMI			0.60	0.60–0.60				−0.52	−0.52–−0.52	
WHtR			0.31	0.28–0.33				−0.34	−0.37–−0.31	
**Girls**	0.89	0.89			0.024	0.86	0.85			0.026
BMI			0.93	0.93–0.94				−0.91	−0.91–−0.91	
WHtR			0.02	0.01–0.03				−0.01	−0.04–−0.01	
**Testing** **IOTF BMI ^b^**										
**Boys**	0.72	0.72			0.035	0.66	0.66			0.037
IOTF BMI										
Thin			−0.13	−0.13–−0.13				0.10	0.10–0.11	
Overweight			0.26	0.25–0.26				−0.23	−0.23–−0.23	
Obese			0.18	0.18–0.19				−0.16	−0.16–−0.15	
Morbid obesity			0.10	0.09–0.11				−0.09	−0.10–−0.08	
WHtR			0.54	0.52–0.57				−0.54	−0.57–−0.52	
**Girls**	0.77	0.77			0.035	0.74	0.73			0.035
IOTF BMI										
Thin			−0.30	−0.31–−0.30				0.30	0.30–0.30	
Overweight			0.30	0.30–0.31				−0.29	−0.29–−0.28	
Obese			0.22	0.22–0.23				−0.22	−0.22–−0.21	
Morbid obesity			0.11	0.10–0.13				−0.11	−0.12–−0.10	
WHtR			0.43	0.40–0.45				−0.02	−0.04–0.01	

Bold represents two alternative models entering either BMI (continuous) alternatively entering IOTF BMI. ^a^ Adjusted for age, maternal education, and city. ^b^ Reference for comparison of IOTF BMI categories set to normal weight.

**Table 3:** Results of backward regression analysis of factors associated with cardiorespiratory fitness (CRF) presented in order of magnitude and comparing models entering FM vs. FFM.

	Unadjusted Model	Fully Adjusted Model ^a^			
	Adj *R*^2^	Adj *R*^2^	F for Model t	*p*<	RSME
**Model: Boys**					
**(Including FM)**	0.17	0.18	81.25	<0.0001	1.11
FM			145.67	<0.0001	
Sports activity			124.88	<0.0001	
Systolic blood pressure			80.49	<0.0001	
WHtR			28.24	<0.0001	
BMI			28.18	<0.0001	
Sedentary activity			10.0	<0.0001	
Consumption of junk food			3.30	0.06	
**Model: Boys**					
**(Including FFM)**	0.16	0.18	67.74	<0.0001	1.11
Sports activity			127.26	<0.0001	
FFM			117.89	<0.0001	
Systolic blood pressure			79.90	<0.0001	
WHtR			33.67	0.02	
BMI			10.72	0.001	
Sedentary activity			9.69	<0.0001	
Consumption of junk food			3.25	0.07	
**Model: Girls**					
**(Including FM)**	0.11	0.12	66.58	<0.0001	1.07
Systolic blood pressure			127.01	<0.0001	
Sports activity			77.67	<0.0001	
FM			60.69	<0.0001	
Consumption of junk food			13.19	0.0003	
BMI			10.37	0.002	
WHtR			4.90	0.02	
**Model: Girls**					
**(including FFM)**	0.11	0.12	61.20	<0.0001	1.08
Systolic blood pressure			129.59	<0.0001	
Sports activity			79.79	<0.0001	
FFM			37.87	<0.0001	
Consumption of junk food			14.61	<0.0001	
WHtR			4.95	<0.0001	
BMI			2.41	0.02	

Bold represents two alternative models entering either FM alternatively entering FFM. ^a^ Adjusted for age, maternal education, and city; BMI = Body Mass Index (continuous); WHtR = Waist–Height Ratio; FM = Percent fat mass; FFM = Percent fat-free mass.

**Table 4:** Adiposity in relation to each cardiometabolic and iron biomarker.

	Unadj. Model ^a^	Adjusted Model ^b^				BMI		WHtR		FM	
	Adj *R*^2^	Adj *R*^2^	F	df	*p* value	Β ^c^	95% CI	β ^c^	95% CI	β ^c^	95% CI
Fasting Glucose											
Boy	0.01	0.06	5.66	10,683	<0.0001	**0.07**	0.05–0.08	0.01	−0.83–0.83	0.01	−0.65–0.62
Girl	0.01	0.11	8.70	10,613	<0.0001	0.01	−0.02–0.05	0.04	−0.02–0.05	0.09	−0.86–0.94
Insulin											
Boy	0.12	0.14	12.05	10,672	<0.0001	**0.16**	0.14–0.17	0.21	−0.84–1.26	0.04	−0.76–−0.84
Girl	0.05	0.08	6.34	10,599	<0.0001	**0.12**	0.07–0.17	0.16	−1.11–1.42	−0.03	−3.07–3.01
Uric Acid											
Boy	0.09	0.13	11.73	10,682	<0.0001	**0.40**	0.36–0.43	0.03	−2.34–2.40	0.21	−2.03–1.61
Girl	0.07	0.09	7.30	10,615	<0.09	**0.77**	0.68–0.86	0.03	−2.26–2.31	0.51	−5.89–4.88
Total Cholesterol											
Boy	0.04	0.04	3.53	10,680	<0.0001	**−0.21**	−0.23–−0.18	0.21	−1.46–1.88	0.11	−1.17–1.39
Girl	0.01	0.02	2.11	10,607	0.02	**0.19**	0.13–0.26	**0.12**	0.12–0.26	0.28	−1.69–1.93
HDL											
Boy	0.04	0.05	4.56	10,681	<0.0001	**−0.28**	−0.29–−0.27	0.10	−0.59–0.79	0.02	−0.55–0.51
Girl		0.03	3.26	10,613	0.03	0.21	−0.66–0.66	−0.11	−0.23–−0.18	0.14	−0.85–0.62
LDL											
Boy	0.03	0.03	3.02	10,669	0.0009	**−0.16**	−0.18–−0.13	0.13	−1.33–1.58	0.16	−0.96–1.28
Girl	0.01	0.01	1.32	10,609	0.21	0.24	−1.40–1.40	0.05	−1.52–1.63	−0.27	−3.97–3.43
Triglycerides											
Boy	0.03	0.03	3.24	10,675	0.0004	0.05	−0.92–1.25	0.16	−0.82–0.86	0.02	−0.15–0.06
Girl	0.06	0.08	6.66	10,610	0.08	**0.32**	0.28–0.36	0.33	−0.70–1.36	0.44	−2.88–1.93
Hemoglobin											
Boy	0.07	0.09	8.15	10,684	<0.0001	**0.31**	0.27–0.34	−0.27	−2.58–2.03	−0.11	−1.87–1.66
Girl	0.00	0.03	3.24	10,612	0.0004	0.05	−2.12–2.12	0.14	−0.04–0.14	0.17	−2.24–2.52
MCHC											
Boy	0.01	0.15	13.68	10,684	<0.0001	0.02	−0.02–0.05	−0.96	−2.46–2.26	−0.08	−1.89–1.73
Girl	0.00	0.15	11.38	10,612	<0.0001	**−0.26**	−0.36–−0.16	0.11	−2.41–2.63	0.11	−5.83–6.06

^a^ Unadjusted models include only predictors: BMI, WHtR, FM; ^b^ models adjusted for age, maternal education, and city; ^c^ Significant β indicated in bold; BMI = Body Mass Index (continuous); WHtR = Waist–Height Ratio; FM = Percent fat mass; HDL= high-density lipoprotein; LDL = low-density lipoprotein; MCHC = mean corpuscular hemoglobin concentration.

## Data Availability

The data are not publicly available due to restrictions set by the funder. Reasonable written requests for basic data will be considered by the Medicover Foundation.

## Supplementary Materials

The following supporting information can be downloaded at: https://www.mdpi.com/article/10.3390/nu14071369/s1, Table S1: STROBE checklist.

## References

[1] Kobza J., Geremek M. (2015). Exploring the Life Expectancy Increase in Poland in the Context of CVD Mortality Fall. INQUIRY J. Health Care Organ. Provis. Financ..

[2] OECD/European Observatory on Health Systems and Policies (2017). Poland: Country Health Profile 2017. State of Health in the EU.

[3] Leon D.A. (2011). Trends in European life expectancy: A salutary view. Int. J. Epidemiol..

[4] Movsisyan N.K., Vinciguerra M., Medina-Inojosa J.R., Lopez-Jimenez F. (2020). Cardiovascular Diseases in Central and Eastern Europe: A Call for More Surveillance and Evidence-Based Health Promotion. Ann. Glob. Health.

[5] Stefler D., Brett D., Sarkadi-Nagy E., Kopczynska E., Detchev S., Bati A., Scrob M., Koenker D., Aleksov B., Douarin E. (2021). Traditional Eastern European diet and mortality: Prospective evidence from the HAPIEE study. Eur. J. Nutr..

[6] Jackson-Leach R., Lobstein T. (2006). Estimated burden of paediatric obesity and co-morbidities in Europe. Part 1. The increase in the prevalence of child obesity in Europe is itself increasing. Int. J. Pediatric Obes..

[7] NCD Risk Factor Collaboration (NCD-RisC) (2016). Worldwide trends in diabetes since 1980: A pooled analysis of 751 population-based studies with 4·4 million participants. Lancet.

[8] Ahluwalia N., Dalmasso P., Rasmussen M., Lipsky L., Currie C., Haug E., Kelly C., Damsgaard M.T., Due P., Tabak I. (2015). Trends in overweight prevalence among 11-, 13- and 15-year-olds in 25 countries in Europe, Canada and USA from 2002 to 2010. Eur. J. Public Health.

[9] Knai C., Suhrcke M., Lobstein T. (2007). Obesity in Eastern Europe: An overview of its health and economic implications. Econ. Hum. Biol..

[10] Chrzanowska M., Koziel S., Ulijaszek S.J. (2007). Changes in BMI and the prevalence of overweight and obesity in children and adolescents in Cracow, Poland, 1971–2000. Econ. Hum. Biol..

[11] NCD Risk Factor Collaboration (NCD-RisC) (2017). Worldwide trends in body-mass index, underweight, overweight, and obesity from 1975 to 2016: A pooled analysis of 2416 population-based measurement studies in 128·9 million children, adolescents, and adults. Lancet.

[12] NCD Risk Factor Collaboration (NCD-RisC) (2020). Height and body-mass index trajectories of school-aged children and adolescents from 1985 to 2019 in 200 countries and territories: A pooled analysis of 2181 population-based studies with 65 million participants. Lancet.

[13] (2015). The Global Strategy for Women’s, Children’s and Adolescents’ Health 2016–2030. Every Woman Every Child. http://www.everywomaneverychild.org/globalstrategy.

[14] Tirosh A., Shai I., Afek A., Dubnov-Raz G., Ayalon N., Gordon B., Derazne E., Tzur D., Shamis A., Vinker S. (2011). Adolescent BMI Trajectory and Risk of Diabetes versus Coronary Disease. N. Engl. J. Med..

[15] Sawyer S.M., Afifi R.A., Bearinger L.H., Blakemore S.-J., Dick B., Ezeh A.C., Patton G.C. (2012). Adolescence: A foundation for future health. Lancet.

[16] Spear B.A. (2002). Adolescent growth and development. J. Am. Diet. Assoc..

[17] Granados A., Gebremariam A., Lee J.M. (2015). Relationship between Timing of Peak Height Velocity and Pubertal Staging in Boys and Girls. J. Clin. Res. Pediatric Endocrinol..

[18] Takamura T., Kita Y., Nakagen M., Sakurai M., Isobe Y., Takeshita Y., Kawai K., Urabe T., Kaneko S. (2017). Weight-adjusted lean body mass and calf circumference are protective against obesity-associated insulin resistance and metabolic abnormalities. Heliyon.

[19] Bel-Serrat S., Ojeda-Rodríguez A., Heinen M.M., Buoncristiano M., Abdrakhmanova S., Duleva V., Sant’Angelo V.F., Fijałkowska A., Hejgaard T., Huidumac C. (2019). Clustering of Multiple Energy Balance-Related Behaviors in School Children and its Association with Overweight and Obesity—WHO European Childhood Obesity Surveillance Initiative (COSI 2015–2017). Nutrients.

[20] Bába B., Ráthonyi G., Müller A., Ráthonyi-Odor K., Balogh P., Ádány R., Bács Z. (2020). Physical Activity of the Population of the Most Obese Country in Europe, Hungary. Front. Public Health.

[21] Sawyer S.M., Azzopardi P.S., Wickremarathne D., Patton G.C. (2018). The age of adolescence. Lancet Child Adolesc. Health.

[22] Barreira T., Staiano A., Katzmarzyk P.T. (2012). Validity assessment of a portable bioimpedance scale to estimate body fat percentage in White and African-American children and adolescents. Pediatric Obes..

[23] Cole T.J., Lobstein T. (2012). Extended international (IOTF) body mass index cut-offs for thinness, overweight and obesity. Pediatric Obes..

[24] Jankowski M., Niedzielska A., Brzezinski M., Drabik J. (2014). Cardiorespiratory Fitness in Children: A Simple Screening Test for Population Studies. Pediatric Cardiol..

[25] Bruggeman B.S., Vincent H.K., Chi X., Filipp S.L., Mercado R., Modave F., Guo Y., Gurka M.J., Bernier A. (2020). Simple tests of cardiorespiratory fitness in a pediatric population. PLoS ONE.

[26] Jacks D.E., Topp R., Moore J.B. (2012). Prediction of VO2 Peak Using a Sub-maximal Bench Step Test in Children. Clin. Kinesiol..

[27] Martinsson A., Andersson C., Andell P., Koul S., Engström G., Smith J.G. (2014). Anemia in the general population: Prevalence, clinical correlates and prognostic impact. Eur. J. Epidemiol..

[28] Alleyne M., Horne M.K., Miller J.L. (2008). Individualized Treatment for Iron-deficiency Anemia in Adults. Am. J. Med..

[29] Andes L.J., Cheng Y.J., Rolka D.B., Gregg E.W., Imperatore G. (2020). Prevalence of Prediabetes among Adolescents and Young Adults in the United States, 2005–2016. JAMA Pediatric.

[30] Rokholm B., Baker J.L., Sørensen T.I.A. (2010). The levelling off of the obesity epidemic since the year 1999—A review of evidence and perspectives. Obes. Rev..

[31] Wang Y., Beydoun M.A., Min J., Xue H., Kaminsky L.A., Cheskin L.J. (2020). Has the prevalence of overweight, obesity and central obesity levelled off in the United States? Trends, patterns, disparities, and future projections for the obesity epidemic. Int. J. Epidemiol..

[32] Schaefer F., Georgi M., Wühl E., Schärer K. (1998). Body mass index and percentage fat mass in healthy German schoolchildren and adolescents. Int. J. Obes..

[33] Suliga E. (2009). Visceral adipose tissue in children and adolescents: A review. Nutr. Res. Rev..

[34] Tumilowicz A., Beal T., Neufeld L.M., Frongillo E.A. (2019). Perspective: Challenges in Use of Adolescent Anthropometry for Understanding the Burden of Malnutrition. Adv. Nutr. Int. Rev. J..

[35] Freedman D.S., Wang J., Maynard L.M., Thornton J.C., Mei Z., Pierson R.N., Dietz W.H., Horlick M. (2005). Relation of BMI to fat and fat-free mass among children and adolescents. Int. J. Obes..

[36] Wang Y. (2004). Epidemiology of childhood obesity—Methodological aspects and guidelines: What is new?. Int. J. Obes..

[37] Wells J.C.K. (2000). A Hattori chart analysis of body mass index in infants and children. Int. J. Obes..

[38] Wells J.C.K., Fewtrell M.S. (2007). Is body composition important for paediatricians?. Arch. Dis. Child..

[39] Kaminsky L.A., Arena R., Beckie T.M., Brubaker P.H., Church T.S., Forman D.E., Franklin B.A., Gulati M., Lavie C.J., Myers J. (2013). The Importance of Cardiorespiratory Fitness in the United States: The Need for a National Registry. Circulation.

[40] Farooq A., Martin A., Janssen X., Wilson M.G., Gibson A., Hughes A., Reilly J.J. (2020). Longitudinal changes in moderate-to-vigorous-intensity physical activity in children and adolescents: A systematic review and meta-analysis. Obes. Rev..

[41] Kwon S., Janz K.F., Letuchy E.M., Burns T.L., Levy S.M. (2015). Developmental Trajectories of Physical Activity, Sports, and Television Viewing During Childhood to Young Adulthood. JAMA Pediatrics.

[42] O’Malley G., Ring-Dimitriou S., Nowicka P., Vania A., Frelut M.-L., Farpour-Lambert N., Weghuber D., Thivel D. (2017). Physical Activity and Physical Fitness in Pediatric Obesity: What are the First Steps for Clinicians? Expert Conclusion from the 2016 ECOG Workshop. Int. J. Exerc. Sci..

[43] Alves A.C., De Silva N.M.G., Karhunen V., Sovio U., Das S., Taal H.R., Warrington N.M., Lewin A.M., Kaakinen M., Cousminer D.L. (2019). GWAS on longitudinal growth traits reveals different genetic factors influencing infant, child, and adult BMI. Sci. Adv..

[44] Ruiter E.L.M., Saat J.J.E.H., Molleman G.R.M., Fransen G.A.J., Van Der Velden K., Van Jaarsveld C.H.M., Engels R., Assendelft W.J.J. (2020). Parents’ underestimation of their child’s weight status. Moderating factors and change over time: A cross-sectional study. PLoS ONE.

[45] Solmi F., Sharpe H., Gage S.H., Maddock J., Lewis G., Patalay P. (2021). Changes in the Prevalence and Correlates of Weight-Control Behaviors and Weight Perception in Adolescents in the UK, 1986-2015. JAMA Pediatrics.

[46] Madsen K.A., Thompson H.R., Linchey J., Ritchie L.D., Gupta S., Neumark-Sztainer D., Crawford P.B., McCulloch C.E., Ibarra-Castro A. (2021). Effect of School-Based Body Mass Index Reporting in California Public Schools. JAMA Pediatrics.

[47] Laird R.D., Pettit G.S., Bates J.E., Dodge K.A. (2003). Parents’ Monitoring-Relevant Knowledge and Adolescents’ Delinquent Behavior: Evidence of Correlated Developmental Changes and Reciprocal Influences. Child Dev..

[48] Parker J.S., Benson M.J. (2004). Parent-adolescent relations and adolescent functioning: Self-esteem, substance abuse, and delin-quency. Adolescence.

[49] Kivimäki M., Kuosma E., Ferrie J.E., Luukkonen R., Nyberg S.T., Alfredsson L., Batty G., Brunner E., Fransson E., Goldberg M. (2017). Overweight, obesity, and risk of cardiometabolic multimorbidity: Pooled analysis of individual-level data for 120 813 adults from 16 cohort studies from the USA and Europe. Lancet Public Health.

[50] Di Bonito P., Valerio G., Licenziati M.R., Campana G., del Giudice E.M., Di Sessa A., Morandi A., Maffeis C., Chiesa C., Pacifico L. (2021). Uric acid, impaired fasting glucose and impaired glucose tolerance in youth with overweight and obesity. Nutr. Metab. Cardiovasc. Dis..

[51] Mireku M.O., Rodriguez A. (2020). Family Income Gradients in Adolescent Obesity, Overweight and Adiposity Persist in Extremely Deprived and Extremely Affluent Neighbourhoods but Not in Middle-Class Neighbourhoods: Evidence from the UK Millennium Cohort Study. Int. J. Environ. Res. Public Health.

[52] Smetkowski M., Płoszaj A., Rok J. (2016). Local Concentration of Deprivation in Poland.

[53] Gomula A., Nowak-Szczepanska N., Danel D., Koziel S. (2015). Overweight trends among Polish schoolchildren before and after the transition from communism to capitalism. Econ. Hum. Biol..

[54] Popławska H., Wilczewski A., Dmitruk A., Hołub W. (2013). The timing of sexual maturation among boys and girls in east-ern Poland, 1980–2000: A rural–urban comparison. Econ. Hum. Biol..

[55] Pikel T.R., Malus T., Starc G., Golja P. (2020). Changes in the Growth and Development of Adolescents in a Country in Socio-Economic Transition 1993–2013. Slov. J. Public Health.

[56] GBD 2019 Risk Factors Collaborators (2020). Global burden of 87 risk factors in 204 countries and territories, 1990–2019: A systematic analysis for the Global Burden of Disease Study 2019. Lancet.

[57] LBD Double Burden of Malnutrition Collaborators (2020). Mapping local patterns of childhood overweight and wasting in low- and middle-income countries between 2000 and 2017. Nat. Med..

[58] Johnson W., William J., Kuh D., Hardy R. (2015). How Has the Age-Related Process of Overweight or Obesity Development Changed over Time? Co-ordinated Analyses of Individual Participant Data from Five United Kingdom Birth Cohorts. PLoS Med..

[59] Ng M., Fleming T., Robinson M., Thomson B., Graetz N., Margono C., Mullany E.C., Biryukov S., Abera S.F., Gakidou E. (2014). Global, regional, and national prevalence of overweight and obesity in children and adults during 1980–2013: A systematic analysis for the global burden of disease study 2013. Lancet.

[60] Skinner A.C., Ravanbakht S.N., Skelton J.A., Perrin E.M., Armstrong S.C. (2018). Prevalence of Obesity and Severe Obesity in US Children, 1999–2016. Pediatrics.

[61] NHS Digital Health Survey for England 2019: Overweight and Obesity in Adults and Children. https://digital.nhs.uk/data-and-information/publications/statistical/health-survey-for-england/2019.

[62] Garrido-Miguel M., Cavero-Redondo I., Alvarez-Bueno C., Rodríguez-Artalejo F., Moreno L.A., Ruiz J.R., Ahrens W., Martínez-Vizcaíno V. (2019). Prevalence and Trends of Overweight and Obesity in European Children From 1999 to 2016. JAMA Pediatrics.

[63] Hanson M., Gluckman P. (2016). Commentary: Developing the future: Life course epidemiology, DOHaD and evolutionary medicine. Int. J. Epidemiol..

[64] Godfrey K., Gluckman P.D., Hanson M. (2010). Developmental origins of metabolic disease: Life course and intergenerational perspectives. Trends Endocrinol. Metab..

[65] Jacob C.M., Baird J., Barker M., Cooper C., Hanson M. (2017). The Importance of a Life Course Approach to Health: Chronic Disease Risk from Preconception through Adolescence and Adulthood.

[66] Khalife N., Kantomaa M., Glover V., Tammelin T., Laitinen J., Ebeling H., Hurtig T., Jarvelin M.-R., Rodriguez A. (2014). Childhood Attention-Deficit/Hyperactivity Disorder Symptoms Are Risk Factors for Obesity and Physical Inactivity in Adolescence. J. Am. Acad. Child Adolesc. Psychiatry.

[67] Brooks S.J., Parks S.M., Stamoulis C. (2021). Widespread Positive Direct and Indirect Effects of Regular Physical Activity on the Developing Functional Connectome in Early Adolescence. Cereb. Cortex.

[68] Eurostat, Births and Fertility in 2016. https://ec.europa.eu/eurostat/documents/2995521/8774296/3-28032018-AP-EN.pdf/fdf8ebdf-a6a4-4153-9ee9-2f05652d8ee0.

[69] Rodriguez A., Miettunen J., Henriksen T.B., Olsen J., Obel C., Taanila A., Ebeling H., Linnet K.M., Moilanen I., Jarvelin M.-R. (2007). Maternal adiposity prior to pregnancy is associated with ADHD symptoms in offspring: Evidence from three prospective pregnancy cohorts. Int. J. Obes..

[70] Rodriguez A. (2010). Maternal pre-pregnancy obesity and risk for inattention and negative emotionality in children. J. Child Psychol. Psychiatry.

[71] Voerman E., Santos S., Golab B.P., Amiano P., Ballester F., Barros H., Bergström A., Charles M.-A., Chatzi L., Chevrier C. (2019). Maternal body mass index, gestational weight gain, and the risk of overweight and obesity across childhood: An individual participant data meta-analysis. PLoS Med..

[72] Kelsey M.M., Zeitler P.S. (2016). Insulin Resistance of Puberty. Curr. Diabetes Rep..

[73] Cousminer D.L., Stergiakouli E., Berry D.J., Ang W., Groen-Blokhuis M.M., Körner A., Siitonen N., Ntalla I., Marinelli M., Perry J.R. (2014). Genome-wide association study of sexual maturation in males and females highlights a role for body mass and menarche loci in male puberty. Hum. Mol. Genet..

[74] Buxton J.L., Das S., Rodríguez A., Kaakinen M., Alves A.C., Sebért S., Millwood I.Y., Laitinen J., O’Reilly P.F., Järvelin M.-R. (2014). Multiple Measures of Adiposity Are Associated with Mean Leukocyte Telomere Length in the Northern Finland Birth Cohort 1966. PLoS ONE.

[75] Henriksson P., Henriksson H., Tynelius M.P., Berglind D., Löf M., Lee M.I.-M., Shiroma S.E.J., Ortega F.B. (2019). Fitness and Body Mass Index During Adolescence and Disability Later in Life: A Cohort Study. Ann. Intern. Med..

[76] Kantomaa M.T., Stamatakis E., Kankaanpää A., Kaakinen M., Rodriguez A., Taanila A., Ahonen T., Järvelin M.-R., Tammelin T. (2012). Physical activity and obesity mediate the association between childhood motor function and adolescents’ academic achievement. Proc. Natl. Acad. Sci. USA.

[77] Dahl R.E., Allen N.B., Wilbrecht L., Suleiman A.B. (2018). Importance of investing in adolescence from a developmental science perspective. Nature.

[78] Cardel M.I., Atkinson M.A., Taveras E.M., Holm J.-C., Kelly A.S. (2020). Obesity Treatment Among Adolescents: A Review of Current Evidence and Future Directions. JAMA Pediatrics.

[79] Patton G.C., Sawyer S.M., Santelli J.S., Ross D.A., Afifi R., Allen N.B., Arora M., Azzopardi P., Baldwin W., Bonell C. (2016). Our future: A Lancet commission on adolescent health and wellbeing. Lancet.

[80] Azzopardi P.S., Hearps S.J.C., Francis K.L., Kennedy E.C., Mokdad A.H., Kassebaum N.J., Lim S., Irvine C.M.S., Vos T., Brown A.D. (2019). Progress in adolescent health and wellbeing: Tracking 12 headline indicators for 195 countries and territories, 1990–2016. Lancet.

